# Beyond 5G Fronthaul Based on FSO Using Spread Spectrum Codes and Graphene Modulators

**DOI:** 10.3390/s23083791

**Published:** 2023-04-07

**Authors:** Daniel Neves, Anderson Sanches, Rafael Nobrega, Hichem Mrabet, Iyad Dayoub, Kohei Ohno, Shyqyri Haxha, Ivan Glesk, Antonio Jurado-Navas, Thiago Raddo

**Affiliations:** 1 Electrical Engineering Department, Federal University of Ceara, Fortaleza 60020-181, Brazil; 2 Engineering, Modeling & Applied Social Sciences Center, Federal University of ABC, Santo Andre 09210-580, Brazil; 3 SERCOM Laboratory, Tunisia Polytechnic School, Carthage University, Carthage 1054, Tunisia; 4 Universite Polytechnique Hauts-de-France, Universite Lille, and INSA Hauts-de-France, 59313 Valenciennes, France; 5 School of Interdisciplinary Mathematical Sciences, Meiji University, Tokyo 101-8301, Japan; 6 Department of Electronic Engineering, Royal Holloway, University of London, London WC1B 5DN, UK; 7 Faculty of Engineering, University of Strathclyde, Glasgow G1 1XJ, UK; 8 Communications and Signal Processing Laboratory, Telecommunication Research Institute, University of Malaga, 29010 Malaga, Spain; 9 Department of Communications Engineering, University of Malaga, 29010 Malaga, Spain

**Keywords:** fronthaul, FSO, 5G, 6G, graphene, modulator, mobile, security, energy, optics

## Abstract

High data rate coverage, security, and energy efficiency will play a key role in the continued performance scaling of next-generation mobile systems. Dense, small mobile cells based on a novel network architecture are part of the answer. Motivated by the recent mounting interest in free-space optical (FSO) technologies, this paper addresses a novel mobile fronthaul network architecture based on FSO, spread spectrum codes, and graphene modulators for the creation of dense small cells. The network uses an energy-efficient graphene modulator to send data bits to be coded with spread codes for achieving higher security before their transmission to remote units via high-speed FSO transmitters. Analytical results show the new fronthaul mobile network can accommodate up to 32 remote antennas under error-free transmissions with forward error correction. Furthermore, the modulator is optimized to provide maximum efficiency in terms of energy consumption per bit. The optimization procedure is carried out by optimizing both the amount of graphene used on the ring resonator and the modulator’s design. The optimized graphene modulator is used in the new fronthaul network and requires as low as 4.6 fJ/bit while enabling high-speed performance up to 42.6 GHz and remarkably using one-quarter of graphene only.

## 1. Introduction

Sixth-generation (6G) telecommunications technologies have recently emerged as a potential candidate to support the vast volume of data traffic required by today’s modern and digital society. Mobile data traffic is growing exponentially every year, along with the number of mobile subscribers worldwide [1]. Mobile systems are expected to handle the increases in global traffic volume, presently doubling every two years [1]. Furthermore, over two billion global 5G users are expected by the end of 2025 [1]. As a consequence, the existing cellular networks will not be able to meet such traffic demands and predicted energy consumption. Next-generation mobile networks will further deal with the changing behavior of high-bandwidth data traffic applications that will eventually be part of the commercial push for new mobile solutions and networking upgrades. Moreover, key performance indicators [2] suggest that beyond 5G mobile systems should support a density of 10 million connections/km${}^{2}$, 500 exabyte/month traffic, and user data rates reaching 1 Gb/s for a few devices and 100 Mb/s for tens of thousands of users highly concentrated in hot-spot areas such as, for example, football stadiums. The densification of mobile cells is a promising way for operators to keep continuously evolving their network’s capacity. The connectivity between central offices (CO) and remote units (RU), which is known as mobile fronthaul, accounts for an important segment of mobile network cells. The creation of ultra-dense networks by deploying mobile cells will play a key role in beyond 5G networks. In fact, this sort of network architecture is expected to reduce the capital and operational costs, as reported in Ref. [3].

Among many fronthaul technologies for deploying a mobile network [4,5,6,7,8,9,10,11,12,13,14,15,16,17,18,19,20,21,22,23,24,25,26,27,28], free-space optics (FSO) have gained momentum mainly due to the progress in the laser-based transmission stage [29]. Laser technologies for high-speed optical telecom are becoming mature, scalable, and cost-efficient [30]. In this fashion, many lasing technologies are commercially available and enabling many interesting networking scenarios. Further, FSO has recently been proposed as a solution for many different emerging applications such as high-speed rail connectivity [31,32,33], high altitude platforms [34], and optical satellite communications [35,36,37] to name a few. In addition, FSO can be seen as a prospective solution for rapid and cost-efficient creation of new mobile cells to support high-bandwidth services where the provision of broadband technologies has not been feasible so far. The elegancy of this wireless technology to create rapid, efficient and temporary broadband services in highly dense areas is quite unique compared to its baseline counterpart based on optical fiber. Although optical fibers are also used in some scenarios of beyond 5G networks, the lack of their deployment flexibility, especially in highly dense urban areas and associated high-costs, pose some real challenges that call for alternative technological solutions [15].

The need for the fast and efficient broadband service provision to support the traffic supply and demand in ultra-dense urban areas grants FSO technologies a potential to become a viable alternative for the fronthaul mobile cells densification. The large interest in FSO [38,39,40,41,42,43,44] is mainly due to its immunity to the radio interference, license-free operation, and optical broadband availability which render this wireless technology as a strong candidate for the deployment of broadband mobile fronthaul systems. FSO has higher security levels than have the main competing technologies such as optical fibers [45,46,47], albeit with some vulnerabilities to eavesdroppers as suggested in Ref. [48]. Security has been a major concern for many applications in communication systems [48]. New industries are becoming digital and being fully connected on an unprecedented scale, increasing the need of cyber resilience and security. In this way, FSO affords the integration along with spread spectrum coding techniques [49,50,51] to support higher security levels. Such combination can be useful against eavesdroppers. Optical code-division multiple-access (OCDMA) is a promising technique to asynchronously transmit multiple users’ signals data over a network in a robust way [52,53,54,55,56,57,58,59]. Data of individual users are encoded before transmission by multiplexing many information channels simultaneously with the use of spread spectrum codes in order to increase user’s security and privacy. Furthermore, OCDMA has several desirable networking features such as symmetric bandwidth support for up- and down-links, soft-capacity on-demand, and burst transmission support. The use of spread spectrum codes can offer a higher security to network users the similar fashion as the wireless CDMA, which was commercially deployed in the 1990’s. FSO based on OCDMA with optical spread codes are regarded as a potent alternative for the next generation mobile networks.

As has already been mentioned, the energy consumption poses real challenges in existing cellular networks. The fronthaul energy consumption is responsible for the large portion of the network operational expenditure (OPEX) [60]. Since the current energy production is still mainly based on fossil sources it directly contributes to a high carbon footprint. Continuous improvements of the mobile fronthaul energy efficiency and reduction in carbon dioxide emissions by means of ‘greener’ technologies have become of major importance to many industries [61]. Therefore the energy efficiency is becoming one of the most important metrics in beyond 5G systems and green technologies will play a pivotal role in the next generation mobile solutions. Reinforcement learning methods also have been used to lower the energy consumption of base stations [62], whereas an evaluation of cellular traffic and communication parameters based on machine learning tools was addressed in Ref. [63]. The power consumption was considered as an optimization problem in Ref. [64], whereas a game-theoretic model is used in Ref. [65]. Lastly, many solutions were proposed in Ref. [66] to cope with energy consumption reduction, but without addressing the hardware solution counterpart.

Energy-efficient data modulators (regardless of the network architecture) will be the key factor in lowering the overall system energy consumption. Optical modulators can be implemented on a combination of different technologies, such as photonics [67], III-V compound [68], barium titanate [69], or graphene [70,71,72]. Nonetheless, existing technologies still face some challenges to meet not only energy-per-bit efficiency, but also bandwidth and footprint needs [73]. Graphene, is a CMOS compatible technology, provides a fast and scalable fabrication environment, with a single layer of carbon atoms, and has many unique physical and chemical properties not yet fully exploited in optical modulators design. The first graphene-based electro-absorption modulator was demonstrated in 2011 [74]. Since then, several graphene integrated modulators have been proposed [70,71,72,74,75,76,77,78]. Graphene-based modulators have already demonstrate their ability to outperform traditional modulators, but there is still plenty of room for optimization [68,72,78,79,80,81]. It has been shown that judicious design and optimization of modulators can enable their unprecedented performance [68,72,82,83,84].

Within this broad context and having in mind the network security and energy efficiency, in this paper, the next-generation fronthaul network architecture based on FSO, spread spectrum encoding techniques, and the use of graphene modulators is proposed and evaluated. The new mobile fronthaul network is fully described. An analytical formalism for evaluating the bit error rate (BER) performance of the proposed network is presented, and the design and optimization of a new optical modulator based on graphene aiming at reducing power consumption is addressed. The modulator’s ring resonator optimization procedure is carried out by changing its graphene content. The optimized graphene modulator is integrated into the new FSO fronthaul network. To the best of our knowledge, this is the first time reporting on the performance of FSO based on a graphene modulator and spread spectrum codes for use in future mobile networks. The main contributions of this paper are summarized as follows:

- Proposed high-speed FSO links for mobile fronthaul networks for cell densification or expansion, where the network footprint is critical and the provision of broadband technologies is poor or not feasible. FSO allows for rapid, efficient and temporary broadband services in highly dense areas in a unique fashion. We integrate FSO along with OCDMA-based coding techniques to enable higher security levels at the physical layer. The system integration of OCDMA and FSO not only improves the transmission capacity and security levels, but also supports multiplexing of many data channels simultaneously via spreading codes to enable a centralized architecture which concentrates the data processing and management at the CO premises. This unique system integration is addressed for the first time:
- Design, optimization, and analysis of a graphene-assisted optical modulator for its use in the FSO fronthaul mobile network. The percentage of graphene used in the modulator design and analysis is varied from 25% to 100% with four discrete steps of 25%. The modulator critical coupling condition representing the OFF-state and a −3 dB level as its ON-state is obtained. Numerical results show that this new modulator consumes as little as 4.6 fJ/bit whilst achieving high-speed operation with bandwidth up to 42.6 GHz when surprisingly using only 25% of graphene;
- Development of a FSO fronthaul network based on a centralized architecture approach for consolidating the signal encoding and modulation stages at the CO premises before signal transmission to antennas at the RU side. Signals are transmitted in an asynchronous fashion by means of assigned spectrum spread codes. The latter grants the system higher security levels at the physical layer as the receiver needs beforehand knowledge of the given code to retrieve the transmitted data bits with success. The BER as well as the signal-to-interference-plus-noise-ratio (SINR) performance of the proposed fronthaul network is addressed by taking into account the FSO, OCDMA, graphene modulator, one-dimensional (1D) codes, avalanche photodiode (APD) receiver, APD noise, thermal noise, and cross-talk among transmitted signals. The optimum threshold of the receiver is also addressed. The best BER network performance is achieved by the 25% graphene modulator among the four analyzed scenarios. Analytical results show that the new mobile network accommodates up to 32 antennas upon error-free transmissions, enabling the creation of ultra-dense small cells or cell expansions.

This paper is organized as follows. Section 2 describes the fronthaul network which is based on a centralized architecture and employs a graphene modulator and spread spectrum techniques before transmission to remote units via FSO transmitters. Section 3 lays out the design and optimization of the graphene modulator. Section 4 presents the analytical framework used to model the FSO signals through the network for BER performance evaluation. Relevant results regarding the proposed network are discussed in Section 5. Finally, Section 6 presents some concluding remarks.

### Related Work

A hybrid FSO and passive optical network (PON) as mobile fronthaul for the development of ultra-dense small cells was proposed in Ref. [27]. Such a hybrid network architecture is interesting, since optical fiber deployment as a fronthaul for every small cell is not feasible due to the high-costs or physical constraints. Nevertheless, the performance evaluation of the network is limited to the latency and considers a time wavelength-division multiplexing scheme which requires end-to-end synchronism throughout the network, which adds complexity to the system. A hybrid network based on FSO and PON was addressed in Ref. [85]. However, such a hybrid network also considers end-to-end signal synchronization, which poses significant challenges in real deployment as any minor time deviation would result in substantial data bit losses at the DU side. Additionally, hybrid FSO and PON rely on existing optical fiber infrastructures that are supposed to be already deployed and available as fiber-to-the-antenna. Accordingly, this suits a limited networking scenario, and it might not be the case for many fronthaul scenarios.

In Ref. [86], a bipolar-OCDMA technique based on spectral amplitude coding is developed for signal transmissions in FSO channels. Further, a hybrid FSO and optical fiber system based on OCDMA was experimentally addressed in Ref. [28]. The system supports 10 Gb/s transmissions and is considered highly secure. However, the system supports only a single link. A network flying platform based on FSO technology for providing services under a vertical fronthaul framework was proposed in Ref. [14]. The platform is particularly interesting for ultra-dense heterogeneous small cells. Nonetheless, the signal synchronization among the elements of the flying platform poses some real complexities and challenges. In Ref. [15], an overview of FSO systems for beyond 5G based on transceiver design and integration with the millimeter wave and terahertz technologies was given. The latter developed a network based on hybrid and mixed FSO, millimeter wave and terahertz technologies. Traditional FSO networks have been demonstrated in several works. For example, a 400 Gb/s FSO was experimentally demonstrated in Ref. [87], while over 800 Gb/s was shown in Ref. [88], and 1 Tb/s was achieved under a single-wavelength FSO system in Ref. [89]. Lastly, a FSO link at 1.28 Tb/s was achieved with the help of multiple wavelengths in Ref. [90]. The integration of multiple wavelengths along with spatial multiplexing resulted in an aggregate bit rate of 14 Tb/s over a 220 m FSO link [91].

Moreover, a FSO transceiver based on a novel, intelligent, lens-based optical beam stabilization technology has recently been demonstrated in Ref. [92]. A new scheme to enhance security based on non-orthogonal multiple access and physical layer coding has been proposed in Ref. [93]. Meanwhile, the uplink transmission of a hybrid FSO and radio frequency network was evaluated against eavesdroppers in Ref. [94]. On the other hand, the feasibility of using FSO technology for flexible architectures of optical fronthaul networks was demonstrated in Ref. [95]. An optical frequency comb for the generation and distribution of low-phase noise signals in 5G fronthaul was used in Ref. [96], but with the main focus on optical distribution networks based on fibers. A recent and comprehensive review of a potential convergence of wireless and optical solutions toward 6G networks was given by Ref. [97].

An experimental performance investigation of a laboratory-controlled FSO setup was carried out in Ref. [98]. The experiments were performed in a chamber for a 5 m link at 10 Gb/s and demonstrated performance improvements when a cyclic redundancy check technique was employed. In contrast, experimental results of a triple-hop relay-based all-optical FSO system with an amplify-and-forward scheme for atmospheric turbulence channels was demonstrated in Ref. [99]. The experiment addressed system channels under different atmospheric regimes for data rates up to 10 Gb/s.

Moreover, a bi-directional fiber-FSO-5G millimeter-wave and sub-THz converged system were demonstrated in Ref. [100]. The system uses an orthogonally polarized dual-carrier to achieve downlink transmissions up to 40 Gb/s/100 GHz while uplink at 10 Gb/s/28 GHz, supporting large traffic capacity. In Ref. [101], the authors experimentally demonstrated a 400 Gb/s FSO mode-division-multiplexing link under turbulence mitigation with adaptive optics by multiplexing four Laguerre–Gaussian modes using both radial and azimuthal indices, each carrying a 50-Gbaud quadrature-phase-shift-keyed signal. Subsequently, in Ref. [102], a 4 Gb/s 16-QAM turbulence-resilient self-coherent FSO link using a pilot tone and an array of photodiodes was demonstrated by the authors. At the transmitter, a Gaussian beam was transmitted with a frequency offset so that the beams have a modal coupling similar to high-order Laguerre–Gaussian modes, while the array of photodiodes increased the overall bandwidth at the receiver side.

Furthermore, the authors experimentally demonstrated a mid-infrared FSO link using wavelength division-multiplexing, mode division-multiplexing, and a combination of both in Ref. [103]. Three wavelengths were multiplexed on a single polarization, with each wavelength carrying two orbital angular momentum beams. Since each beam carries 50 Gb/s QPSK data, the system achieved a total capacity of 300 Gb/s, which represents an increase of around 30 times (∼30×) compared to the single channel and beam link in Ref. [104]. By its turn, a FSO link at 10 Gb/s using a unipolar quantum opto-electronic system at room temperature was demonstrated in Ref. [104].

Moreover, the authors experimentally demonstrated an adaptive multi-modal FSO transceiver to increase the robustness of FSO communications under atmospheric turbulence in Ref. [105]. Accordingly, by applying time-division multiplexing techniques combined with a spatial light modulator under the turbulence effect, an aggregate data rate of 590 Gb/s for five transmit modes and two polarizations was successfully achieved. In contrast, the authors experimentally demonstrated the mitigation of atmospheric turbulent effects in an FSO system with a 3 × 1 MISO (multi-input single-output) transmitter based on OAM modes in Ref. [106]. They showed the feasibility of the system operating under intensity-modulated direct-detection transmitting three OAM modes with 39.06 Gb/s over a 1.2 m free-space link at 1550 nm. Lastly, the authors experimentally demonstrated a single multi-plane light convertor to mitigate turbulence-induced crosstalk and demultiplexed channels for a 1550 nm to FSO link with two OAM multiplexed channels, each carrying a 100 Gb/s-QPSK signal in Ref. [107].

## 2. Novel Network Architecture Design

**Figure 1:**
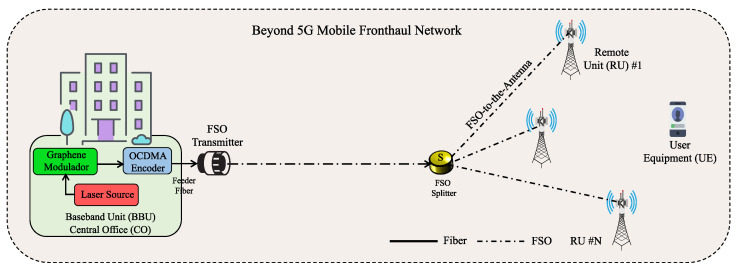
Architecture of the mobile fronthaul network based on FSO, graphene modulator, and spread spectrum codes. FSO: free-space optics. OCDMA: optical code-division multiple-access.

Mobile fronthaul networks support a large number of antennas over small mobile cells to increase data rates, coverage, and data traffic support. As more and more 5G networks roll out [1], energy-efficient fronthaul technologies are becoming a desired answer for improving the networks’ energy consumption and OPEX [60]. As an example, the power consumption of a (micro) cell can reach up to 300 kW [108]. Thus, future generation of networks will therefore need to increasingly rely on energy-efficient multiplexing schemes, network devices, and disruptive approaches. The proposed next-generation FSO is one such approach. By using the FSO approach, one can simultaneously transmit data signals to many antennas from a centralized location via FSO-to-the-antenna, as illustrated in Figure 1.

The network consists of data transmission bits between the BBU at the CO and a cell site RU, where the centralized BBU serves multiple RU. Several RUs are connected to the BBU with a high-speed transport network, that is, the FSO fronthaul interface, which can support data rates up to several gigabits. This significantly improves the economy of scaling of the fronthaul networks’ design. In fact, this approach becomes particularly interesting for FSO technology when transmission of an optical signal can be realized from a fiber termination point over the free-space channel without the need for any additional complexity. Afterwards, at the receiver side, the signal is directly coupled into an optical fiber. Thus, the need to convert the signal from electrical-to-optical or vice versa for transmitting or receiving through the free-space channel is removed. The mobile fronthaul accommodates a pool of BBU at the CO premises, whereas the RU at the tower site becomes simpler (in design, complexity, and lower cost) and can be implemented based on a straightforward radio frequency (RF) front-end antenna [109]. This centralized architecture eventually grants additional flexibility and reduces overall power consumption of the system [110]. The adoption of centralized fronthaul avoids the need for modulation/demodulation stages at the RU site, which contributes to energy consumption reduction. Accordingly, the optical carrier is modulated with RF data signals at the CO premises to be transmitted over the FSO channel to many antennas. The modulation is carried out by means of a graphene-based modulator as part of the optoelectronic integrated BBU capability [2,111]. This modulation happens in intensity, as supported by the proposed graphene modulator, which will be addressed in the next section. At the transmitter side, a single-wavelength laser source is modulated by an ON–OFF keying technique using a novel graphene modulator to generate bits. Sequentially, this optical signal is coded by an OCDMA encoder [49]. The spread spectrum technique is used to increase the security of data transmissions. This process is repeated for a given number of antennas or RU.

The spread spectrum technique extends the original data bandwidth over a broader bandwidth via a “spreading the code” sequence. In this way, an exclusive code sequence with weight *W* and length *L* is assigned to each signal granting data codification at the physical layer. OCDMA coding techniques offer a certain level of security and built-in privacy, since the OCDMA receiver can only retrieve the transmitted data with knowledge of the employed code. Since the original signal is spread using a pseudo-random code, that is, the signal appears random-like, it becomes more resilient against eavesdropping. The total number of simultaneous users, thus transmitted signals, in the network is *N*; the code’s chip duration for all users is $T_{c}=T_{b}/L$, where $T_{b}$ is the bit period. After users’ data encoding, the resulting signals are recombined in optical fiber and fed into an FSO transmitter (see Figure 1). The FSO transmitter via the FSO splitter sends over the free space the signals to the individual RU. However, signals are transmitted over a single channel that gives rise to a cross-talk known as multi-access interference (MAI). MAI affects the receiver of the RU and is known to limit the number of simultaneous signals and the achievable BER. It is noteworthy that the OCDMA encoding procedure does not modify the signal’s phase, but only the signal intensity, since it is based on an incoherent coding approach.

**Figure 2:**
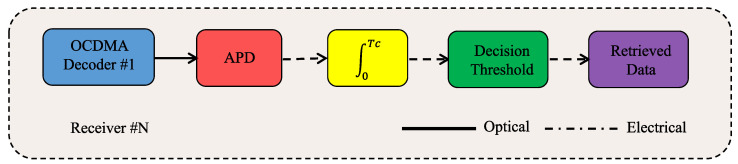
Block diagram of the decoder at the receiver side showing the OCDMA decoder, the avalanche photodetector (APD), the integrator with $T_{c}$ as chip duration, the threshold decision device, and recovered data.

Lastly, at the receiver side, the FSO receiver collects the transmitted RF signal. The OCDMA decoder, as illustrated in Figure 2, decodes the received signal by removing the temporal translation introduced by the encoder, and thus realigns the signal into a single pulse called an OCDMA correlation peak. The decoder’s output is directly detected by an avalanche photodetector (APD), where its electrical output signal is then integrated over the chip period, and finally compared to a threshold level by a threshold decision device, and the transmitted data bit is finally retrieved. The APD and thermal noises as well as the MAI are all considered during the system’s BER performance assessment. The focus is also on the cross-talk among the transmitted signals, generally considered to be a dominant noise source upon OCDMA coding techniques [49,57]. It is worth mentioning, without any loss of generality, that due to the expected signal levels, the non-linear impairments are neglected. Lastly, it is worth mentioning that the choice for APD receivers is due to the proposed network scenario, which may trigger random signal fluctuations at the receiver due to the orientation/positioning deviation of the antennas at the RU premises [112,113,114]. Hence, the APD receiver is a better choice over traditional PIN receivers, as it grants more flexibility to the system [112,113,114,115].

## 3. Graphene Modulator for Next-Generation Fronthaul Networks

### 3.1. Design, Optimization, and Numerical Simulations

**Figure 3:**
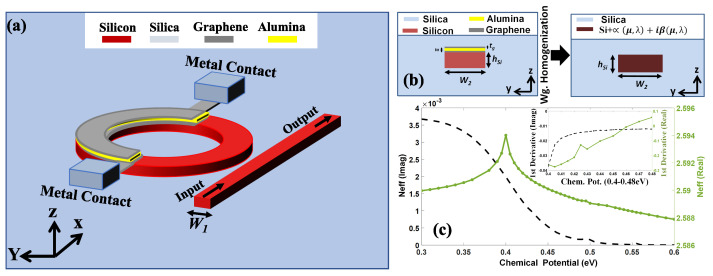
(**a**) Schematic representation of the homogenization approximation technique for the resonator waveguide. (**b**) Real (green) and imaginary (black) part of an effective index for the graphene modulator. Inset: first derivative of real (green) and imaginary (black) parts of refractive index for 0.4–0.48 eV. (**c**) Front view of a graphene ring-resonator coupled with a bus waveguide.

The design of the proposed modulator is also illustrated in Figure 3. The modulator is based on a silicon core bus waveguide having a width of $W_{1}=340$ nm coupled to a ring-shaped silicon resonator of a fixed waveguide width of $W_{2}=600$ nm. Both waveguides have the same thickness of $h_{Si}=220$ nm. Moreover, the resonator is partially covered with three thin layers. The first layer is composed of graphene and has a thickness of $h_{g}=0.34$ nm, followed by an alumina layer with a thickness of $h_{a}=7$ nm, and finally another graphene with $h_{g}=0.34$ nm. The whole structure is on the silica substrate and is more propitious for footprint reduction due to its double-layered structure [72].

The device also has two metal contacts on the top of the silica substrate, each connected to a graphene layer, forming a capacitor. Such a device can be eventually fabricated using chemical vapor deposition, photolithography, and etching. To simplify the simulation procedure but without losing any accuracy, both layers of graphene and the alumina one are replaced by a homogeneous waveguide, with the refraction index $n_{homog}$ given as $n_{homog}=n_{Si}+\alpha \left(\mu ,\lambda \right)+j\beta \left(\mu ,\lambda \right)$ and $neff_{graphene}=neff_{homog}$, where α(μ,λ) and β(μ,λ) are the real and imaginary parts of the silicon refractive index being a function of the wavelength and chemical potential, respectively. Here, $n_{Si}$ is the silicon refractive index, and $neff_{homog}$ and $neff_{graphene}$ are the effective refraction index of the homogeneous and graphene-assisted waveguides, respectively [78]. In particular, this method renders a homogeneous waveguide instead of a classical heterogeneous waveguide with many layers, and consequently, several different indexes of refraction, with an estimated error of ∼0.05% only. Subsequently, the chemical potential properties of the modulator for different amounts of graphene are analyzed. A schematic illustration of the waveguide homogenization technique is depicted in detail in Figure 3a. The variation of the real and imaginary parts of the index versus the chemical potential for a wavelength of 1.5344 μm is plotted in Figure 3b.

The modulator operates in the OFF state with a chemical potential of 0.444 eV, which represents the highest value from the optimal range of the graphene’s real and imaginary conductivity (see Figure 3). This range maximizes the modulator’s optical absorption and phase variation ratio, reducing power consumption. The ON state of the modulator is obtained when the insertion loss is −3 dB. The analysis was repeated for different amounts of graphene, ranging from 25% to 100% in four increments, with a ring’s external radius of 2.08 μm. Accordingly, the modulator demonstrates a maximum modulation depth of 27 dB at 1534.4 nm, with a ring’s external radius of 2.08 μm, and graphene and alumina layers of 0.34 nm and 7 nm. Next, the device’s bandwidth is given by Ref. [82]

(1) $$BW_{-3\;\mathrm{dB}}\left(\mathrm{nm}\right)=\frac{\left(1-\alpha^{2}\right)\lambda_{0}^{2}}{2\pi^{2}Rn_{g}\alpha },$$

where $n_{g}$, *R*, and $\lambda_{0}$, are the group index, mean radius, and resonance wavelength of the resonator for the propagated mode, respectively. It is considered the same criterion of insertion loss for the ON state (−3 dB) and identical external radius for different amounts of the graphene’s ring percentage. By using the values obtained via numerical simulations for the chemical potential for each graphene amount, the power consumption per bit (Joules/bit) will be, in which $\rho_{ON}>\rho_{OFF}$,

(2) $$E_{\frac{J}{bit}}=\frac{1}{2}\frac{\left(Q_{ON}^{2}-Q_{OFF}^{2}\right)}{2C}=\frac{1}{4}\left(\rho_{ON}^{2}-\rho_{OFF}^{2}\right)\frac{e^{2}h_{diel}S}{\varepsilon_{0}\varepsilon_{r}},$$

where $Q_{ON}$ and $Q_{OFF}$ represent the amount of charge that correspond to the ON and OFF states, *C* is the modulator’s capacitance, $\rho_{ON}$ and $\rho_{OFF}$ are the carrier concentrations for the graphene’s chemical potential equivalent to the ON and OFF states, *e* is the electron charge, $h_{diel}$ is the dielectric thickness, $\varepsilon_{0}$ and $\varepsilon_{r}$ are the vacuum and relative permittivity, respectively, and *S* represents the capacitor’s area, which corresponds to the area on the top surface of the resonator. Lastly, the −3 dB bandwidth of the proposed graphene modulator is addressed. To do so, a figure-of-merit (FOM) of the modulator is defined as [82]

(3) $$FOM=\frac{FWHM(GHz)}{Power\;Consumption\left(\frac{E}{bit}\right)\times Area\left(\mu m^{2}\right)},$$

where FWHM is the full width at half-maximum pulse in the frequency domain, *E* is the energy required for switching, and PowerConsumption and Area are the power-per-bit consumption and full area of the device, respectively. The FOM provides an expression of the efficiency of the device, that is, a measure of its capabilities and performance. This is valuable for optimization of the design of modulators. Careful design and optimization of the parameters can provide an incredible degree of freedom in the modulator’s features, resulting in singular performances. Then, the optimization procedure is carried out by optimizing the modulator’s design for both geometric and physical parameters. The parameters along with some performance metrics are summarized in Table 1. Note that $\mu_{OFF1}$ is the initial value of the chemical potential for the OFF state, whereas $\mu_{OFF2}$ is the value after optimization. In a similar fashion, $Loss_{1}$ is the total resonator loss (bending and absorption losses) before the optimization procedure, and $Loss_{2}$ is the optimized value. The same procedure is applied for the remaining parameters. The optimization renders a more energy-efficient device with a larger bandwidth (due to lower $\mu_{OFF}$ and $\mu_{ON}$).

### 3.2. Numerical Simulations and Results

**Figure 4:**
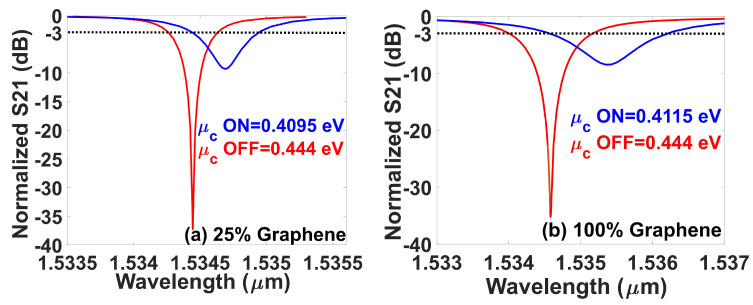
The modulator’s transmission curves versus the wavelength considering both OFF (red) and ON (blue) states and different percentages of graphene. (**a**) At 25% graphene and (**b**) 100% graphene.

**Figure 5:**
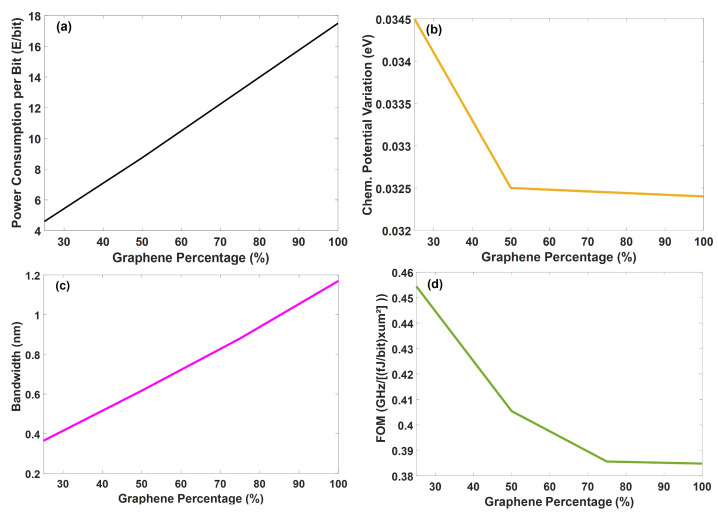
The performance of the modulator. (**a**) The power consumption per bit versus the graphene percentage. (**b**) The chemical potential variation versus the graphene percentage. (**c**) The −3 dB-bandwidth versus the graphene percentage. (**d**) The trade-off performance efficiency.

The new graphene modulator’s performance was evaluated for four different graphene levels: 25%, 50%, 75%, and 100%. Only four discrete steps were considered due to the computational demand of the simulations. The goal was to determine the modulator’s maximum efficiency in terms of power consumption per bit. Initially, we carried out an analysis of the graphene modulator properties considering the transmission parameter ($S_{21}$). The transmission parameter versus wavelength considering both OFF and ON states has been plotted in Figure 4 for 25% and 100% graphene only, for the sake of space. It can be noted that both curves (red and blue) have a minor displacement of the propagation wavelength. This happened due to the insignificant phase variation in comparison to the propagation wavelength. The latter occurred owing to the substantially smaller device’s coupling propagation region than the beat length [82]. The extinction ratios are 34.27 dB, 39.13 dB, 51.52 dB, and 32.26 dB for 25%, 50%, 75%, and 100% graphene. Let us analyze how the device’s power consumption performs under different amounts of graphene used in the ring. The goal is to identify the more efficient approach that simultaneously ensures modulation within the GHz range whilst providing power efficiency. Thus, the power consumption per bit as a function of the graphene’s ring percentage is shown in Figure 5a. Notice that the optical losses related to the graphene’s absorption rapidly increase the power consumption per bit and are proportional with the graphene’s ring percentage in the modulator. Although the power consumption remains within targeted levels, the modulation capacity of the device can be better understood by analysing the difference between the chemical potentials for the states ON and OFF. Then, the chemical potential ON–OFF variation as a function of the graphene’s percentage was plotted in Figure 5b. Interestingly, these results provide clear evidence on how much graphene should be used. The modulator shows a decrease in the chemical potential ON–OFF variation as a function of the graphene ring percentage.

The −3 dB bandwidth as a function of the ring’s graphene content levels in percentages is plotted in Figure 5c. Results show that the bandwidth increases significantly with the graphene ring percentage. This occurs due to increases of graphene-related losses as the graphene percentage is increased. Consequently, the modulator can offer ultra-high switching properties if higher percentages of graphene are used. Nevertheless, the 100% graphene modulator has 6.72 μm${}^{2}$, although the 25% graphene modulator has an active area of 1.68 μm${}^{2}$. This represents an effective 4-fold decrease in the modulator’s active area. There is also a cost decrease along with the footprint reduction. Bear in mind that superior-standard graphene might be costly. The final cost of the modulator depends on the quantity of graphene used among others, with the 25% version being significantly cheaper than the 100% version. Lastly, the performance efficiency of the modulator as a function of the graphene’s ring percentage is investigated. These results are shown in Figure 5d. Note the overall performance deteriorates by increasing the ring’s graphene percentage. In this case, the reduction in the active resonator area via the increases of the graphene in ring broadening percentage renders a substantial reduction in the efficiency of the device. This happens since the large bandwidth obtained by the increasing graphene ring effect is compensated by the deleterious effect of the high-power consumption per bit. This indicates that, for the sake of minimizing power consumption, the quantity of graphene should be capped at a quarter of the total cavity, at the cost of reducing the bandwidth. Such a device can be built to fit an extremely small area of 22.1 μm${}^{2}$ and still offer a wide bandwidth of 42.6 GHz with very low levels of power consumption, such as 4.6 fJ/bit.

Next, the RC constant is addressed. Initially, the contact resistance equals to 100Ω·μm for each graphene’s plate capacitor (Ni/graphene contact). The plate capacitance equals to 12.48 fF/μm${}^{2}$[71]. Then, according to the model for calculating $f_{RC}$ (RC limited 3 dB bandwidth) [71], the quantum capacitance for a device with an active area of 1.68 μm${}^{2}$ (25% of graphene) is $C_{Q}=0.87$ pF, while the plate capacitance is $C_{P}=21.25$ fF. Accordingly, being the equivalent capacitance value of the circuit, we have $C=\left(1/2\right)\left(C_{Q}C_{P}\right)/\left(0.5C_{Q}+C_{P}\right)=20.26$ fF. The equivalent resistance of the circuit is given by $R=2(R_{g}+R_{c})$, where $R_{g}$ is the electrical resistance associated with the graphene layer, and $R_{c}$ is the resistance of the graphene–metal interface. Considering that $R_{g}$ is small for devices with dimensions in the order of a few microns [71], $R_{c}$ becomes dominant. Additionally considering how the contact length between the graphene monolayer and electrode is represented by the graphene length around the resonator circumference, the experimental values of the contact resistance equivalent to the proposed device equal to $R_{c}=35.77\;\Omega$. Finally, given the optical bandwidth of 42.6 GHz, the calculation of the modulation bandwidth is given by $1/f_{-3\;dB}^{2}=1/f_{RC}^{2}+1/f_{opt}^{2}\approx 42.67$ GHz. Therefore, $f_{-3\;dB}$ is close to the value associated with $f_{opt}$ (optical limited 3 dB bandwidth) and $f_{RC}$ (RC limited 3 dB bandwidth) presents reasonable values and does not limit $f_{-3\;dB}$ (total 3 dB modulation bandwidth given by $f_{opt}$ and $f_{RC}$) for the proposed modulator device. The same analysis holds true for different percentages of graphene used in the device design. Accordingly, the value of $f_{RC}=110$ GHz, regardless of the device’s active area. Nonetheless, the values of *R* and *C* change with the given active area. For example, for an active area of 1.68 μm${}^{2}$ (25% of graphene) R=71.54Ω and C=20.26 fF, for 3.36 μm${}^{2}$ (50% graphene) R=35.77Ω and C=40.52 fF, for 5.04 μm${}^{2}$ (75% graphene) R=23.84Ω and C=60.78 fF, and for 6.72 μm${}^{2}$ (100% graphene) R=17.88Ω and C=81.04 fF, respectively.

## 4. Network Performance Evaluation

In this section, a closed-form analytic expression for the bit error rate associated to the FSO fronthaul based on the OCDMA and graphene modulator is derived. The coded-FSO mobile fronthaul network employs an on–off keying (OOK) intensity-modulated incoherent structure, where each signal is transmitted via a code sequence for data bit “1”, whereas no signal is transmitted for data bit “0”. Despite the fact that several noise sources can affect the performance of the fronthaul network, the main signal’s degradation source considered here is the multiple interference among signals, that is, cross-talk which is the dominant noise in such systems [52,57]. Additionally considered are the APD noise and thermal noise at the receiver side. The 1D code employed in the network has a maximum non-zero shift auto-correlation and cross-correlation bounded by one, and each interfering signal from the cross-talk contributes with only one chip overlapping on the desired signal’s code. The signals’ transmissions are assumed as chip-synchronous, which reflects the worst possible scenario for the performance analysis. Although the transmission of signals through a free-space channel might be affected by time-varying inhomogeneities in the atmosphere refractive index, this work focuses on a new fronthaul network architecture and its overall performance assessment for the sake of simplicity. Readers interested in the performance evaluation of FSO systems in the presence of atmospheric turbulence as well as pointing errors at the receiver side should read Refs. [39,49]. Nonetheless, a new analytical formalism considering atmospheric turbulence as well as pointing errors will be developed and published elsewhere as future work.

Then, the OOK modulation format and optical orthogonal codes (OOC) are applied to modify the information signals to be more conveniently transmitted over the channel. In this case, each information bit from the *n* signals is coded into a code sequence as

(4) $$s_{n}=Pb_{n}\left(t\right)c_{n}\left(t\right),\;\;\;\;\;\;\;\;0\leq t\leq T=FT_{c},$$

and

(5) $$c_{n}\left(t\right)=\sum_{j=0}^{F-1}c_{n}\left(j\right)P_{T_{c}}\left(t-jT_{c}\right),\;\;\;\;\;\;\;\;n=1,2,3,...N,$$

where *P* is the power transmitted by the graphene modulator, $b_{n}\left(t\right)\in \left\{0,1\right\}$, and $c_{n}\left(j\right)\in \left\{0,1\right\}$ are the value associated to the user’s information bit and OOC chips for the *n*-th user, respectively, *L* is the code length, and $P_{T_{c}}\left(t-jT_{c}\right)$ is a unit rectangular pulse of duration $T_{c}$. At the receiver’s side, assuming *N* active signals transmitting signals in the network, the received optical field at the decoder output of each antenna user will be

(6) $$r\left(t\right)=\sum_{n=1}^{N}s_{n}\left(t-\tau_{n}\right),$$

where $\tau_{n}$ is the relative network transit delay of the *n*-th antenna. This delay accounts for the lack of synchronization between transmitters. It is also considered chip synchronization, a situation that does not take advantage of the OCDMA coding technique concept where completely asynchronous traffic, either of bits or chips, is possible [58]. Nonetheless, this assumption greatly simplifies the formalism, and the obtained expression for the BER reflects the worst possible scenario. Sequentially, the received signal r(t) is multiplied by a stored replica of desired users for a single-bit transmission per sequence period. The probability that a given number of photons is absorbed from an incident optical field by the APD receiver over a chip interval is given by a Poisson distribution. The photon absorption rate due to a mark transmission in the desired user antenna code can be written as [116]

(7) $$\lambda_{s}=\frac{\eta \lambda }{hc}P,$$

where η is the APD efficiency, λ is the optical wavelength, *h* is Planck’s constant, and *c* is the speed of light in vacuum. The receiver integrates the APD output over the code symbol interval $T=LT_{c}$. Hence, the integral APD output of the desired user antenna is

(8) $$Z_{1}=\frac{1}{T_{c}}\int_{0}^{T}APD\left(r\left(t\right)c_{1}\left(t\right)-n_{b}\left(t\right)\right)dt.$$

By considering the occurrence of data bits “0” and “1” to be independent variables and that their probability density functions (PDF) can be represented by Gaussian functions [49], then, the mean and variance of the error probabilities conditioned to decision variables of the desired user antenna when it transmits the bit “0” and when $I_{1}$ interfering signals are active in the channel will be [117]

(9) $$\mu_{b0}=GT_{c}\left[I_{1}\lambda_{s}+\frac{\left(WN-I_{1}\right)\lambda_{s}}{M_{e}}+L\left(\lambda_{b}-\frac{I_{b}}{e}\right)\right]+LT_{c}\frac{Is}{e},$$

and

(10) $$\sigma_{b0}^{2}=G^{2}F_{e}T_{c}\left[I_{1}\lambda_{s}+\frac{\left(WN-I_{1}\right)\lambda_{s}}{M_{e}}+L\left(\lambda_{b}+\frac{I_{b}}{e}\right)\right]+L\left(T_{c}\frac{I_{s}}{e}+\sigma_{th}^{2}\right),$$

where *G* is the APD gain, $F_{e}=k_{eff}G+\left(2-1/G\right)\left(1-k_{eff}\right)$ is the excess noise factor, $k_{eff}$ is the APD effective ionization ratio, *W* is the OOC code weight, $M_{e}$ is the modulation extinction ratio, $\lambda_{b}$ is the background light photon arrival rate, $I_{b}$ is the APD bulk leakage current, $I_{s}$ is the APD surface leakage current, $\sigma_{th}^{2}=2k_{B}T_{R}T_{c}/e^{2}R_{L}$ is the thermal noise variance, $k_{B}$ is Boltzmann’s constant, $T_{R}$ is the receiver noise temperature, and $R_{L}$ is the receiver load resistor. The value of the receiver parameters is shown in Table 2. Moreover, the mean and variance of the error probabilities conditioned to decision variables of the desired user antenna when it transmits the bit “1” will be

(11) $$\mu_{b1}=GT_{c}\left[\left(W+I_{1}\right)\lambda_{s}+\frac{\left(WN-\left(W+I_{1}\right)\right)\lambda_{s}}{M_{e}}+L\left(\lambda_{b}+\frac{I_{b}}{e}\right)\right]+LT_{c}\frac{I_{s}}{e},$$

and

(12) $$\sigma_{b1}^{2}=G^{2}F_{e}T_{c}\left[\left(W+I_{1}\right)\lambda_{s}+\frac{\left(WN-\left(W+I_{1}\right)\right)\lambda_{s}}{M_{e}}+L\left(\lambda_{b}+\frac{I_{b}}{e}\right)\right]+L\left(T_{c}\frac{I_{s}}{e}+\sigma_{th}^{2}\right).$$

Further, the PDF of $I_{1}$ (total undesired interference contribution to for a single-bit transmission to the desired receiver’s accumulated output at the correlation time) can be written as [116]

(13) pI1(i)=∑i=0N−1N−1ipiqN−1−iδI1−i,

where $p=W^{2}/2L$, q=1−p, and δ(x) is the Dirac delta function. Because the receiver chooses a “1” if $Z_{1}>T_{h}$, and chooses “0” otherwise, the optimum receiver uses the value of $T_{h}$ which minimizes the overall bit error probability. Then, the average bit error probability is given by

(14) $$\begin{array}{cc} P_{b}\left(Error\right) & =\min_{T_{h}}E_{I_{1},b}\;\left[P_{b}\left(Error|I_{1},b,T_{h}\right)\right]\\ \; & =\min_{T_{h}}\frac{1}{2}\left[1+\sum_{i=0}^{N-1}pI_{1}\left(i\right)\left\{Q\left(\frac{T_{h}-\mu_{b0}\left(i\right)}{\sigma_{b0}\left(i\right)}\right)+Q\left(\frac{T_{h}-\mu_{b1}\left(i\right)}{\sigma_{b1}\left(i\right)}\right)\right\}\right]. \end{array}$$

## 5. Numerical Results of the Network

Here, the analytical formalism developed in the previous section is applied to the performance evaluation of the FSO-coded fronthaul network supporting the creation of dense antenna cells via the OCDMA multiplexing technique. The BER and SINR (signal-to-interference-plus-noise-ratio) performances of the network where each of 32 simultaneous signals is assigned a unique 1D-OCDMA code is evaluated. The 32 different codes were generated on a single wavelength beforehand with a code length L=1000 and weight W=10. However, one can also aim to achieve different data rate transmissions via different configurations of the graphene modulator. This is possible because the different amounts of changes in graphene directly affect both the extinction ratio and modulator’s speed, consequently affecting the bandwidth of the modulated signals. As already mentioned, four configurations holding amounts of 25%, 50%, 75%, and 100% of graphene are considered. These structures achieve extinction ratios of the order of 34.27 dB, 39.13 dB, 51.52 dB, and 32.26 dB with bandwidth values of 42.6 GHz, 63.81 GHz, 78.41 GHz, and 88.45 GHz, respectively. Consequently, the data rate transmissions performed for such configurations are 60.2 Gb/s, 90.2 Gb/s, 110.9 Gb/s, and 125.1 Gb/s, respectively. The main values of the parameters of the fronthaul network are summarized in Table 2. With all common configurations of the graphene modulators properly described, the performance of the coded-FSO fronthaul network can now be addressed. For the sake of simplicity and conciseness, the network segment from the RU to the user equipment (see Figure 1) is not considered in the performance analysis.

**Figure 6:**
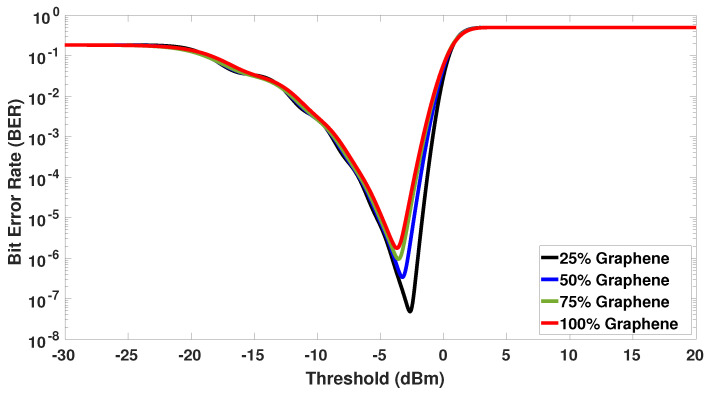
Optimum detection receiver threshold. The BER is minimized by choosing a threshold value of −2.5 dBm for the 25% graphene modulator.

It is noteworthy that (14) has been successfully validated against the BER equation given by (5) in Ref. [118]. Initially, the BER is investigated as a function of the threshold level to be adjusted at the receiver side of the coded-FSO fronthaul network for all the graphene modulators (i.e., 25%, 50%, 75%, and 100% graphene). The final stage at the receiver side is responsible for choosing between the bit transmission “1” or “0” if the decision variable (electrical power in the device input) is greater or lower than the threshold, respectively. Hence, it is necessary to estimate an optimum threshold that minimizes the BER once the bit levels are dependent on the cross-talk among signals and photodetector noise levels in the coded-FSO fronthaul network evaluated. In this scenario, the optical receiver power is set to 0 dBm and the number of simultaneous antennas is fixed at 32. Accordingly, the BER versus the threshold level is plotted in Figure 6. Initially, it can be observed that the optimum threshold to minimize the BER increases as the percentage of graphene decreases. This is simply because more photons (or optical power) arrive at the photodetector as both the extinction ratio and bit rate transmission related to the signals are not too high. Results suggest that the optimum thresholds are −2.66 dBm, −3.27 dBm, −3.58 dBm, and −3.70 dBm for modulators with 25%, 50%, 75%, and 100% graphene, respectively. In addition, it can be observed that the modulator with 25% graphene has the lowest BER at the optimum threshold among all the configurations. It occurs because the cross-talk is the main deleterious source in the coded-FSO fronthaul network once the photodetector noises are negligible at high levels of received optical power [49,116]. Lastly, it is possible to further note that the bit error probability approaches 1/2 as the threshold decreases or increases from the optimum point. The low levels of thresholds result in erroneous decisions of the bits “0” to “1” even as low levels of cross-talk and photodetector noises upon construction interfere with the signals. In contrast, high thresholds are harmful because most of the bits “1” are interpreted as “0”, even when low levels of photodetector noises perform on the signals.

**Figure 7:**
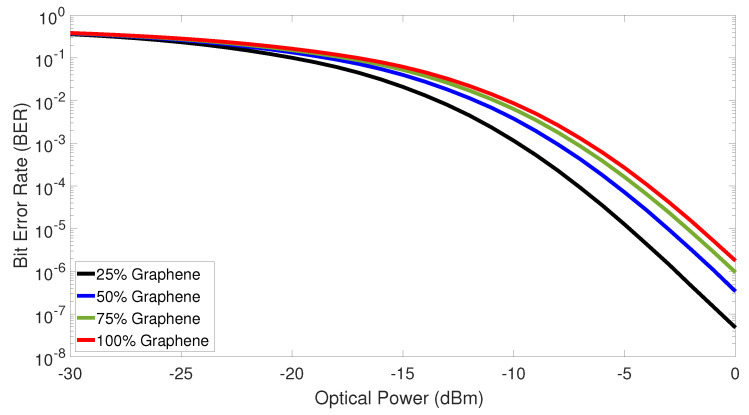
Bit error rate versus the optical power received in the photodetector for different amounts of graphene in the modulator.

Next, the BER versus the optical power for different modulator’s graphene percentage was plotted in Figure 7. In general, the BER improves as the optical power levels increase. In such analyses, we considered 32 simultaneous antennas in the coded-FSO fronthaul network, and also the optimum threshold values as previously obtained. As can be seen, the curves nearly overlap as low optical power reaches the receiver. Such behavior is due to the coded-FSO fronthaul network performance becoming limited to the photodetector noises. In contrast, a considerable difference among the graphene modulators’ performance can be realized in higher optical powers once the cross-talk among the transmitted signals becomes most relevant degradation mechanism. One can still notice that the 25% graphene modulator has the best BER among the different graphene percentages due to its higher modulation extinction ratio. One can further note both 75% and 100% have similar levels of BER. At higher levels of graphene, the modulators have similar higher modulation extinction ratios and bandwidths, consequently achieving similar low BER performances. Bear in mind that the 100% graphene modulator supports a higher bandwidth than does 25% graphene. Nonetheless, both the lower modulation extinction ratio and bandwidth of the 25% graphene modulator leads to better BER performance due to the increased signal power that reaches the photodetector.

**Figure 8:**
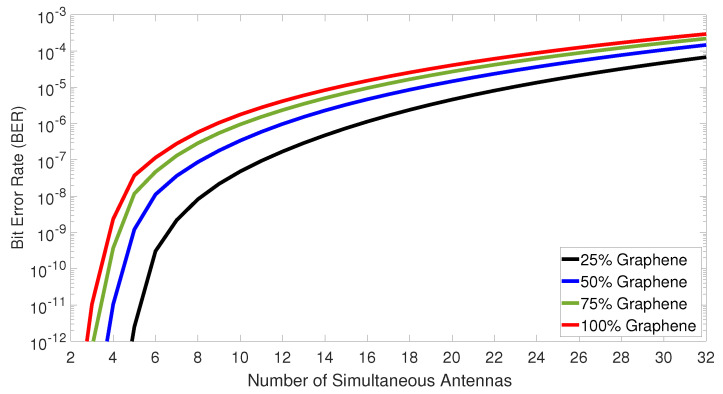
Bit error rate versus the number of simultaneous antennas at the remote unit side.

Subsequently, the BER versus the number of simultaneous antennas was plotted in Figure 8. The number of simultaneous antennas was varied, considering that each one receives 0 dBm of optical power. Additionally, the optimum thresholds were estimated dynamically and employed at the receivers for each group of simultaneous antennas. It needs to be noted that the 25% graphene modulator has the best overall performance. The latter can accommodate up to 11 simultaneous antennas at the mobile cell under BER $\leq 10^{-7}$, and up to 6 under BER $\leq 10^{-9}$. Finally, if someone is interested in accommodating 32 simultaneous antennas under an error-free regime, where all data bits are transmitted with success, FEC techniques at higher levels of BER (i.e., BER $\leq 10^{-3}$) can be deployed [58]. For example, under FEC usage (BER $\leq 10^{-3}$), all investigated graphene modulators can achieve error-free transmissions, supporting the creation of ultra-dense small cells, or cell expansion.

**Figure 9:**
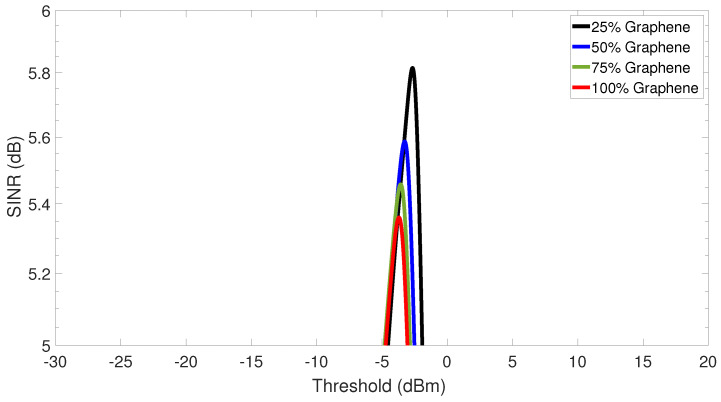
Signal-to-interference-plus-noise-ratio (SINR) versus the optimum threshold.

Next, the SINR as a function of the optimum receiver threshold is plotted in Figure 9 for four different percentages of the modulator’s graphene. To do so, the optical receiver is set to 0 dBm and the number of simultaneous antennas is fixed at 32, for the sake of consistency of the analysis. Bear in mind that this is the SINR at the receiver side after signal detection. One can note from Figure 9 that similar threshold levels are obtained for the four different amounts of graphene in the system’s modulator. Results suggest that the optimum threshold is nearly −2.66 dBm, −3.27 dBm, −3.58 dBm, and −3.70 dBm for the modulator’s configuration with 25%, 50%, 75%, and 100%, respectively. Finally, it can be further observed that the 25% graphene modulator has a slightly higher SINR than the remaining modulator configurations.

**Figure 10:**
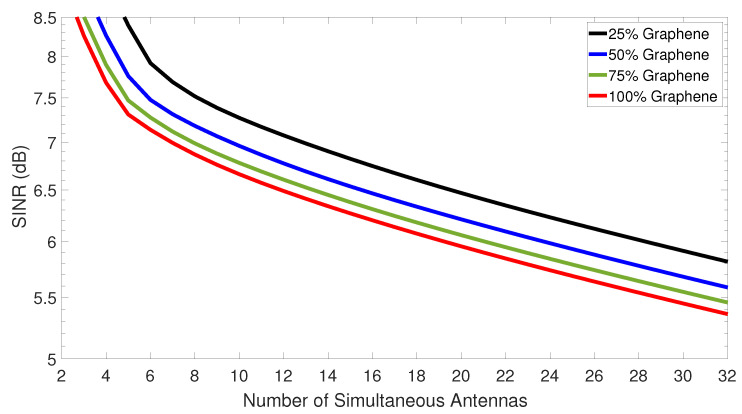
Signal-to-interference-plus-noise-ratio (SINR) versus the number of simultaneous antennas at the remote unit side.

Afterwards, the SINR versus the number of simultaneous antennas was plotted in Figure 10 for four different amounts of the modulator’s graphene. It can be seen that the modulator upon 25% graphene stands out and shows the best performance among the four configurations of graphene. Hence, the SINR follows a similar trend in the performance analysis, where the overall system performance can be impacted by the different amounts of graphene, while 25% shows the best performance. Lastly, one can further note from Figure 10 that the SINR degrades as the number of simultaneous antennas in the mobile cell increases, regardless of the modulator’s graphene amount. This can happen due to the interference raise in the channel associated with the accommodation of more simultaneous antennas in the mobile cell.

## 6. Conclusions

In this paper, a new fronthaul network architecture based on the FSO, the graphene modulator implementation, and spread spectrum coding techniques was proposed, analyzed and evaluated. The newly proposed network architecture employs the FSO instead of traditional wired approaches in order to transmit data bits to the network antennas at the RU side. It is important to note that this approach is also viable for regions that lack wired solutions based on fiber or copper wiring. Here, the signals to be transmitted are first modulated with the novel energy-efficient graphene modulator and multiplexed using the OCDMA spread-spectrum technique. An analytical formalism for performance assessment of the proposed network is presented. The formalism accounts for 1D codes used for carrying users’ signals, and APD and thermal noises in the receiver, cross-talk, and OOK modulation also account for different configurations of the graphene modulator. The modulator optimization was aimed at reducing its power consumption and was carried out by changing the amount of graphene in the modulator ring resonator by varying it from 25% to 100% in four discrete steps. Numerical results show the new graphene modulator consumes as little as 4.6 fJ/bit at 42.6 GHz when containing 25% graphene. This novel 42.6 GHz modulator not only reduces the overall power consumption, but also reduces its overall cost (the 25% graphene modulator is cheaper to produce than the 100% one). Furthermore, the optimum receiver threshold to minimize the BER performance of the network was also determined. Analytical results show that the best BER performance of all four considered modulator configurations can be achieved just by using 25% of graphene. The new FSO fronthaul network can also simultaneously accommodate up to 32 remote antennas with error-free data transmissions upon using FEC. Such a network solution can be interesting under scenarios such as the provision of secure broadband services, ultra-dense small cell creation, or cell expansions. To the best of the authors’ knowledge, this paper is the first report on the design and performance analyses of power-efficient, highly secure FSO networks for use in the future mobile fronthaul.

## Figures and Tables

**Table 1:** Optimization of the modulator’s design and performance metrics.

Graphene (%)	25	50	75	100
$\mu_{{\mathrm{OFF}}_{1}}$ **(eV)**	0.51	0.51	0.51	0.51
$\mu_{{\mathrm{OFF}}_{2}}$ **(eV)**	0.444	0.444	0.444	0.444
$\mu_{{\mathrm{ON}}_{1}}$ **(eV)**	0.468	0.474	0.4815	0.485
$\mu_{{\mathrm{ON}}_{2}}$ **(eV)**	0.4095	0.4115	0.4115	0.4115
${\mathrm{BW}}_{3{\mathrm{dB}}_{1}}$ **(GHz)**	18.1	22	25.1	28.6
${\mathrm{BW}}_{3{\mathrm{dB}}_{2}}$ **(GHz)**	42.6	63.8	78.4	88.45
${\mathrm{Gap}}_{1}$ **(nm)**	275	260	250	240
${\mathrm{Gap}}_{2}$ **(nm)**	205	170	145	125
${\mathrm{Loss}}_{1}$ **(dB/μm)**	−0.0035	−0.0043	−0.0055	−0.0056
${\mathrm{Loss}}_{2}$ **(dB/μm)**	−0.0089	−0.015	−0.0214	−0.0285
$E/{\mathrm{bit}}_{1}$ **(fJ/bit)**	8.4	14.7	17.8	21
$E/{\mathrm{bit}}_{2}$ **(fJ/bit)**	4.6	8.75	13.1	17.5
${\mathrm{FOM}}_{1}$ $\left(\frac{\mathrm{GHz}}{\mathrm{fJ}/\mathrm{bit}\times \mu m^{2}}\right)$	0.0972	0.0677	0.0639	0.0616
${\mathrm{FOM}}_{2}$ $\left(\frac{\mathrm{GHz}}{\mathrm{fJ}/\mathrm{bit}\times \mu m^{2}}\right)$	0.4184	0.3302	0.2704	0.2237

**Table 2:** FSO network parameters.

Parameter	Symbol	Value
Laser wavelength	λ	1550 nm
APD efficiency	η	0.85
APD gain	*G*	100
APD effective ionization ratio	$k_{eff}$	0.02
APD bulk leakage current	$I_{b}$	0.1 nA
APD surface leakage current	$I_{s}$	10 nA
Background light photon arrival rate	$\lambda_{b}$	$10^{9}$ electrons/s
Modulation extinction ratio	$M_{e}$	depends on % graphene
Bandwidth	*B*	depends on % graphene
Data bit rate	$R_{b}=\frac{\sqrt{2}}{2}B=\frac{1}{T}$	depends on % graphene
Receiver noise temperature	$T_{r}$	1100 K
Receiver load resistor	$R_{L}$	1030 Ω

## Data Availability

Not applicable.
